# Revisiting the Warburg Effect: Diet-Based Strategies for Cancer Prevention

**DOI:** 10.1155/2020/8105735

**Published:** 2020-08-04

**Authors:** Quangdon Tran, Hyunji Lee, Chaeyeong Kim, Gyeyeong Kong, Nayoung Gong, So Hee Kwon, Jisoo Park, Seon-Hwan Kim, Jongsun Park

**Affiliations:** ^1^Department of Pharmacology, College of Medicine, Chungnam National University, Daejeon 35015, Republic of Korea; ^2^Department of Medical Science, Metabolic Syndrome and Cell Signaling Laboratory, Institute for Cancer Research, College of Medicine, Chungnam National University, Daejeon 35015, Republic of Korea; ^3^College of Pharmacy, Yonsei Institute of Pharmaceutical Sciences, Yonsei University, Incheon 21983, Republic of Korea; ^4^Department of Life Science, Hyehwa Liberal Arts College, Daejeon University, Daejeon 34520, Republic of Korea; ^5^Department of Neurosurgery, Institute for Cancer Research, College of Medicine, Chungnam National University, Daejeon 35015, Republic of Korea

## Abstract

It is widely acknowledged that cancer cell energy metabolism relies mainly on anaerobic glycolysis; this phenomenon is described as the Warburg effect. However, whether the Warburg effect is caused by genetic dysregulation in cancer or is the cause of cancer remains unknown. The exact reasons and physiology of this abnormal metabolism are unclear; therefore, many researchers have attempted to reduce malignant cell growth in tumors in preclinical and clinical studies. Anticancer strategies based on the Warburg effect have involved the use of drug compounds and dietary changes. We recently reviewed applications of the Warburg effect to understand the benefits of this unusual cancer-related metabolism. In the current article, we summarize diet strategies for cancer treatment based on the Warburg effect.

## 1. Introduction

Unlike normal differentiated cells, which rely primarily on mitochondrial oxidative phosphorylation to generate the energy needed for cellular processes, most cancer cells rely on aerobic glycolysis, in a phenomenon termed the Warburg effect. In the 1920s, Warburg discovered enhanced oxygen uptake and subsequent rapid cell division upon fertilization; he hypothesized that cancer cells might also take up more O_2_ than normal cells [1]. In 1956, Warburg reported that cancer cells exhibit high rates of glucose uptake and lactic acid production, even in the presence of oxygen [2], with cancer cells appearing to prefer aerobic glycolysis to oxidative phosphorylation (OXPHOS). Warburg also initially suspected impaired respiration in cancer cells due to functional defects in mitochondria [3]; however, findings from his own laboratory [4] and those of others [5, 6] indicated otherwise. The mitochondria were found to be necessary for tumor growth [7]. However, cancer glycolysis produces only two moles of ATP per one mole of glucose [8]. This context is still controversial. In many cancers, aerobic glycolysis is upregulated without mitochondrial dysfunction (no identifiable mitochondrial gene mutations) or OXPHOS disruption [9–11]. In these cancers, OXPHOS continues normally, producing as much ATP as OXPHOS in normal tissue under the same oxygen pressures [9–11]. In a new model of cancer metabolism, Kim reported that cancer cell mitochondria exhibit active oxidative phosphorylation [8]. NADH production from glutamine in the cytosol plays a key role of ATP production through the mitochondrial electron transport chain in cancer cells, while NADH production is mostly occupied inside the mitochondria in normal cells [8]. This hypothesis contradicts Warburg's claim for mitochondrial defects in cancer, but after more than half a century of research, Warburg's observations have been applied to most cancer cells, becoming the seventh feature of cancer cells: (1) persistent growth signals, (2) evasion of apoptosis, (3) insensitivity to antigrowth signals, (4) unlimited replicative potential, (5) angiogenesis, and (6) invasion and metastasis [12–14]. Aerobic glycolysis has also been observed in rapidly proliferating normal cells such as stimulated lymphocytes and mitotic and proliferating fibroblasts [15–20], suggesting the association of aerobic glycolysis and rapid growth and proliferation. Upregulation of glycolysis occurs not only in ATP synthesis but also in the synthesis of biomass, including ribonucleotide [21] and nicotinamide adenine dinucleotide phosphate (NADPH) production [22], which can remove reactive oxygen species (ROS) generated by accelerated cancer cell metabolism under hypoxic conditions [22, 23]. Thus, the Warburg effect appears to be strategically driven by cancer cells, while they simultaneously meet several urgent requirements for proliferation in an ever-changing microenvironment under numerous material limitations such as the lack of oxygen and nutrients and proper control of ROS production.

The exact reason and physiological value of abnormal metabolism in cancer should still be revealed. The Warburg effect is generally thought to confer growth advantages to tumor cells including the rapid supply of ATP, amino acids for protein synthesis, nucleic acids for DNA duplication, and lipids for cell biomembrane synthesis, which may be needed in cell proliferation. These processes generate an acidic environment, which is harmful to normal cells but has no effect to tumor cells [24]; fewer ROS are produced, such that the cancer cell genome may elude damage due to a high ROS concentration, leading to apoptosis resistance in tumor subjects [25, 26]. The Warburg effect is now more attractive to scientists. The cause of the Warburg effect caught the attention of scientists, because understanding the cause of the Warburg effect can make more effective treatment for cancer. Indeed, numerous studies have proposed different models of the Warburg effect, which may lead to the identification of its underlying mechanism. In addition, some anticancer drugs have been developed by using the transition from oxidative phosphorylation to glycolytic metabolism in cancer [27], besides diagnosis and detection of metastasis by using F-18 fluorodeoxyglucose- (FDG-) positron emission tomography (PET).

## 2. Targeting Metabolic Mediators

Previous reviews have described several compounds that mediate characteristics of the Warburg effect [28] including (i) increased expression of glucose transporters and thus increased glucose uptake; (ii) increased pentose phosphate pathway-catalyzed NADPH production; (iii) altered activity of glycolytic or glycolysis-related enzymes such as hypoxia-inducible factors/MYC-induced activation of hexokinase 2, lactate dehydrogenase A, and pyruvate dehydrogenase kinase-1 and the switch from pyruvate kinase isozymes M1 to the less active pyruvate kinase isozymes M2; and (iv) increased lactate production. Some of these characteristics have been or could potentially be targeted to develop cancer therapeutics (Table 1, [28]). For example, inhibiting glucose transport slows glucose supply to cancer cells, slowing cancer metabolism and biomass synthesis. As a result, cancer cells fail to grow and instead undergo apoptosis [29]. Many glucose transporter (GLUT) inhibitors have been previously studied [30, 31]. The principle and the use of 2-deoxy-D-glucose (2-DG), dichloroacetate (DCA), and 3-bromopyruvate (3-BP) in cancer therapy has also been addressed in our previous review (Table 2, [28]). Targeting Warburg effect mediators is thus emerging as a promising strategy for cancer treatment.

## 3. Modulating Metabolite Flow

### 3.1. Less Is More: Calorie Restriction (CR) and Cancer Therapy Response

Proposed in 1914, CR was the first method offered for cancer prevention by reducing tumor blood supply [32]. The impact of CR on cancer suppression has since been replicated in studies of brain, prostate, and breast tumors [33–40]. The effect of CR on cancer prevention is based on the Warburg theory, by regulating several metabolic mediators. The reduction of lower circulating glucose, in turn, lowers insulin levels and increases transcription of insulin-like growth factor binding protein- (IGFBP-) 1, consequently decreasing the bioavailability of insulin-like growth factor-1 (IGF-1) [41]. By binding to specific tyrosine kinase receptors, insulin and free IGF-1 activate the phosphatidylinositol-3 kinase- (PI3K-) protein kinase B- (PKB-) mammalian target of rapamycin complex 1 (mTORC1) pathways, which promote proliferative signaling, protect against cell death, and alter cellular metabolism including increased fermentation of glucose and glutamine [42]. CR activates the nuclear factor erythroid 2-related factor 2 (Nrf2) gene [43], an energy-sensing network consisting of adenosine monophosphate-activated protein kinase (AMPK), NAD-dependent deacetylase sirtuin-1 (SIRT1) [44], peroxisome proliferator-activated receptor-alpha (PPAR*α*), and peroxisome proliferator-activated receptor gamma coactivator 1-alpha (PGC1*α*), which counteract the insulin/IGF-1/PI3K/PKB/mTORC1 pathway and promote mitochondrial function.

**Figure 1:**
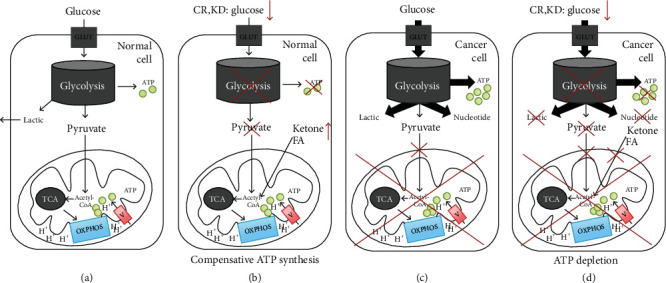
Principles of calorie restriction (CR) and ketogenic diet (KD) in cancer therapy by targeting the Warburg effect. Metabolic differences of normal cells versus cancer cells. (a) In normal cells, once glucose is uptaken into the cells by GLUT, it subsequently enters the glycolysis, generating ATP and pyruvate. The pyruvate then entered the mitochondria and is catalyzed to acetyl-CoA, a substrate of the TCA cycle. Products from the TCA cycle provide substrates for OXPHOS complexes thereby providing a necessary ATP amount via ATP synthase (complex V). (b) In case of normal cells feeding into CR or KD, glucose lever is low, and the glycolysis and ATP from this process are prevented. However, the enhancement of ketone level by CR or KD could still stabilize acetyl-coA level in the mitochondria thus compensating the needed ATP. (c) As mentioned in the Warburg theory, cancer cells trigger large glucose uptake and glycolysis, which provide enough ATP, nucleotide, and lactic acid for cancer growth. (d) CR and KD abolish glycolysis, resulting in reduced needed biomass materials such as nucleotide and microenvironment such as lactic acid. Moreover, mitochondrial dysfunction and lack of mitochondrial necessary enzymes metabolizing ketone bodies to acetyl-coA cause the mitochondria to not generate to compensate for ATP. Thus, cancer could not proliferate probably. Taken together, using CR or KD can specifically target cancer growth. (Thin arrows represent normal stimulation/activation; thick arrows represent overstimulation/activation).

Interestingly, metabolic responses to CR differ between normal and cancer cells. Effectively, CR shuts off the energy source of the cancer. As described earlier, normal differentiated cells rely primarily on mitochondrial oxidative phosphorylation to generate the energy needed for cellular processes, whereas cancer cells rely on aerobic glycolysis. In normal cells, abundant acetyl-CoA from the breakdown of ketone bodies (acetoacetate and *β*-hydroxybutyrate) and fatty acids due to starvation inhibits glycolysis to ensure stable ATP levels, and subsequent oxidation of ketone bodies in peripheral tissue decreases the NADP^+^/NADPH ratio [45]. Mitochondria in tumor cells were initially thought to be dysfunctional [3, 13]. However, several cancer cells also lack the mitochondrial enzymes necessary to metabolize ketone bodies [46–49]. Theoretically, the drop in glycolytic ATP production achieved by CR cannot be compensated for via oxidative phosphorylation; thus, ATP depletion, cell growth inhibition [50], and death ensue [51–53]. Although cancer mitochondrial dysfunction is still under debate, CR has great potential as a cancer therapy simply because of glycolysis depletion, eliminating the advantages conferred to tumor cells by the Warburg effect such as the synthesis of biomass including ribonucleotides [21], amino acids [54], and NADPH [22] (Figures 1(c) and 1(d)).

To date, many studies of the tumor-suppressive effects of CR have focused on its ability to prevent cancer, as an intervention rather than as an application as an anticancer therapy. Recent interest has focused on the potential of CR as an adjunct therapy for various cancers, in tandem with traditional chemotherapy or radiation therapy. CR increases radiation efficacy in breast cancer [55]. Similarly, fasting-based intervention has been demonstrated to protect normal cells while keeping cancer cells vulnerable to high-dose chemotherapy in both cell culture and neuroblastoma-bearing mice [56–59]. However, it remains unknown whether these observations are caused by the Warburg effect.

### 3.2. The Emerging Role of Ketogenic Diets (KDs) in Cancer Treatment

In a KD, fats account for about 90% or more of total energy intake. The KD simulates fasting, increasing ketones in the blood and reducing glucose; fatty acid oxidation and acetyl-CoA production are also increased at high rates. KDs have recently attracted attention, with a broad-spectrum approach aimed at lowering blood sugar and insulin levels, targeting the Warburg effect and fundamental genetic changes [60]. This dietary approach exploits the main metabolic differences between micronutrient loss or limitation, mimicking fasting to some extent by lowering and stabilizing insulin levels, mildly elevating cortisol levels, and raising fatty acid oxidation. Together, these adaptions promote hepatic ketogenesis, raising the concentration of ketones. KDs are therefore attractive for long-term application during cancer treatment. Many studies have revealed pleiotropic effects of KDs on malignant tumors due to changes in systemic and cellular metabolism (Table 1).

Thus, the basis for providing a fat-rich and low-carbohydrate diet in cancer therapy is to lower circulating glucose levels and cause ketosis, which depletes the energy of cancer cells, while normal cells use ketone bodies through metabolism. Reducing blood sugar also reduces the levels of insulin and insulin-like growth factors, which are important drivers of cancer cell proliferation. Due to the Warburg effect, glucose in dietary carbohydrates acts as a primary metabolic fuel for many tumors. This observation prompted early research into KD as a cancer treatment, and carbohydrate restriction-induced glucose deprivation was thought to be the main mechanism by which KD slows tumor progression. KD and CR target the same molecular pathways including PI3K, PKB, mTORC, and AMPK. Several preclinical models have reported that ketosis is associated with tumor growth inhibition either by direct action or as an indicator of the effect of maximal insulin inhibition [61–64]. KD has been shown to delay human gastric cancer cell growth in nude mice [65] and in a xenograft model of prostate cancer [66]. Changes in gene expression suggest that KD can inhibit the IGF-1, platelet-derived growth factor (PDGF), and epidermal growth factor receptor (EGFR) signaling pathways, as shown in various CR and KD studies [34, 53, 67].

### 3.3. Clinical Experience in Humans

The first clinical attempts to control tumor growth by reducing glucose supply to cancer cells were performed in 1987 [68], after the original concept was developed by Gold [69] and by Ray et al. [70]. These researchers found that hydrazine sulphate administration significantly reduced amino acid flux and may favorably influence metabolic abnormalities in cancer cachexia.

However, recent nutritional therapy approaches have been tailored to tumor metabolic properties. In 2005, Breitkreutz et al. [71] investigated gastrointestinal carcinomas in a randomized trial of 23 moderately malnourished patients and found that a high-fat diet may support the maintenance of both body weight and body cell mass, while decreasing lymphocyte numbers; several aspects of quality of life were rated as improved by patients consuming a fat-enriched artificial liquid diet. Although these results do not indicate direct tumor reduction by diet, they may offer a supportive strategy for cancer therapy.

Since the first known applications of KDs to target the Warburg effect specifically were published in 1941/1942, there has been only sporadic interest in KDs for cancer treatment [60, 72]. Clinical results have included a case report of two female pediatric patients with advanced-stage malignant astrocytoma, who demonstrated a 21.8% decrease in glucose uptake at the tumor site when fed a KD, as determined by FDG–PET [73]. Although a KD diet does not replace conventional antineoplastic treatments, these preliminary results suggest that potential clinical application of KDs merits further research.

A recent case report showed improvement in a 65-year-old female patient with glioblastoma multiform treated with a CR-KD, together with standard treatment [74]. Studies of cancer patient quality of life have found that KDs produce no serious side effects, improve emotional functioning, and reduce insomnia [75]. At present, 62 trials are being performed to evaluate low-carbohydrate diets as potential therapies for various diseases, among which 11 are evaluating KDs as adjuvant cancer therapies. At Würzburg University in Germany, KDs are being tested in patients for whom traditional cancer treatment has failed and no other remedy options remain. Preliminary reports indicate that these patients were able to continue the KD therapy for over 3 months and showed improvement, including stable physical condition, tumor shrinkage, and/or slowed tumor growth [75].

Most studies have suggested that CR and KD combined with other clinical therapies can improve cancer treatment. The clinical evidence for CR and KD curing cancer as a conventional anticancer treatment is very poor and is a subject of continuing study (NCT00575146). Previous clinical studies of CR and/or KD treatment of cancer patients are summarized in Table 3. Overall, these studies show that CR and KD are safe for long periods in cancer patients.

## 4. Conclusion and Perspectives

In the presence of oxygen, normal cells undergo glycolysis and oxidative phosphorylation, whereas proliferating cancer cells exhibit an increased glucose uptake and glycolysis rate and, predominantly, undergo lactic acid fermentation. The physiology of cancer metabolism remains to be elucidated, although several drug compounds and therapeutic strategies have been proposed for cancer treatment based on the Warburg effect, which is generally thought to confer growth advantages to tumor cells by increasing ATP and biomass production, generating tumor-promoting environments by increasing lactic acid [24], and decreasing ROS production [25, 26]. Therefore, anticancer strategies are being developed to eliminate these benefits to cancer cells. To target cancer metabolic processes, diet-based strategies can be used in pushbike with drug treatments as an emerging and promising cancer therapy. CR and/or KD can starve cancer cells, while maintaining normal cells. CR and KD reduce glucose levels, eliminating the benefits of glycolysis to cancer cells. These dietary strategies enhance ketones and other metabolites that normally interact with the mitochondrial ATP generation process. OXPHOS was initially thought to be dysfunctional in cancer cells, which lack the mitochondrial enzymes necessary to metabolize ketone bodies.

Theoretically, CR and KD can completely remove the ATP sources of cancer cells but not those of normal cells. These strategies have been applied in both preclinical and clinical studies; however, much stronger effects on cancer proliferation are required to cure cancer. It is necessary to determine whether cancer mitochondria are actually dysfunctional, as well as the exact role of such abnormal mitochondria in cancer cell function. Other aspects, is there any side effect caused by KD? Is there any impairment in rapidly proliferating normal cells (lymphocytes, fibroblasts) that have active aerobic glycolysis [15–20]? Moreover, in a study, Ozsvari et al. demonstrated that mitoketoscins, novel mitochondrial inhibitors for targeting ketone metabolism, could inhibit cancer stem cell activity and propagation [76]. Thus, could KD block cancer stem cell itself? Addressing these questions is critical for the development of new diet-based tools for the improvement of recent therapies by targeting cancer cell metabolism.

## Figures and Tables

**Table 1:** Mechanisms for antitumor effects of the ketogenic diet.

Physiological change	Mechanisms for antitumor effect
Reduce insulin level and signaling	Lower insulin levels reduce oncogenic signaling pathways: PI3K-PKB-mTOR, RAS-RAF-MAPK.
Decrease blood glucose	Glucose restriction sensitizes tumor cells to radiotherapy and chemotherapy.
Enhance fatty acids and ketone bodies	Preclinical inhibition of glycolysis through fatty acids and ketone bodies (Randle cycle) is problematic for tumor cells with dysfunctional mitochondria that rely on glycolysis for energy and antioxidant production.
Increase *β*-hydroxybutyrate	*β*-Hydroxybutyrate is an endogenous histone deacetylase inhibitor with the potential to epigenetically alter protein expression in tumors towards a less aggressive phenotype.
Increase decanoic acid (if medium chain triglyceride oil is part of the ketogenic diet)	Decanoic acid is a PPARg agonist and inhibits AMPA glutamate receptors, which are overexpressed by human glioblastoma cells.

Adapted from [77].

**Table 2:** Therapeutics targeting the Warburg effect in cancers.

Target	Compound	Effect	Status	References
GLUT1	WZB117, STF-31	Inhibits CLUT1 Induces cell cycle arrest and inhibits cancer cell growth	Preclinical	[30, 31]
HK	2DG	Inhibits HK Tolerable adverse effects	Clinical trials discontinued	[78]
PKM2	TEPP-46	Activates PKM2 Tetramer formation and suppress tumorigenesis	Preclinical	[79] [80]
LDHA	FX11	Inhibits LDHA Oxidative stress and inhibits tumor progression	Preclinical	[81]
G6PD	6-AN	Induces oxidative stress Induces cell cycle arrest and apoptosis selectively in irradiated human malignant cells	Preclinical	[82]
MCT1	AZD3965	Inhibits uptake of extracellular lactate	Phase I	[83]
PDK1	DCA	Inhibits PDK1	Phase I-II	[84] [85]
PKB	AZD5363	Inhibits PKB activity	Phase I-II	[86]
	GDC0068		Phase I	[87]
	GSK2141795		Phase I completed	[88]
	GSK2110183		Phase I-II completed Phase II	[88]
	MK-2206	Akt inhibitor enhances antitumor efficacy by standard chemotherapeutic agents or molecular targeted drugs in vitro and in vivo	Phase I-II	[89]

Adapted from [90].

**Table 3:** Clinical studies with CR/KD in cancer patients.

Cancer	Study group	Diet	Outcome	References
Malignant astrocytoma tumors	2	KD > 85 kcal/kg/d (12 mos) > 88 kcal/kg/day (8 wks)	↓PET, 1 patient alive at 4 years and 1 at 10 years	[73]
A partial gastrectomy and total colectomy for familial adenomatous polyposis	1	Parenteral nutrition 28 kcal/kg/d carbohydrates 45 g (5 months)	Treatment well tolerated	[91]
Mix: breast, lung, prostate, ovary…	10	CR (20-140 h pretherapy) (8-56 h posttherapy)	Low chemotherapy side effects	[92]
Glioblastoma	1	Patient conducted water-only therapeutic fasting and a restricted 4 : 1 (fat : carbohydrate+protein) ketogenic diet that delivered about 600 kcal/day	Complete response with radio chemotherapy	[74]
Mix: ovarian, breast, thyroid…	16	KD (less than 70 g carbohydrates per day) 3-month intervention period	1/3 completed CR, 3/4 tolerated well, few side effects from CR	[75]
Mix: breast, lung, colorectum, ovary…	10	KD 17 kcal/kg/d (4 wks)	Level of ketosis (not weight loss) correlated with tumor response	[93]
Advanced stage	Pediatric patients	Medium chain triglyceride- (MCT-) based KD (60% MCT, 20% protein, 10% carbohydrate, and 10% other dietary fats)	Blood ketone levels increased 20- to 30-fold; blood glucose levels declined	[94]
Gastrointestinal tract	27	Parental feeding with lipid-based diet (80% of total caloric requirement were fat, 20% dextrose) or glucose-based diet (100% dextrose)	Number of replicating cells increased in average 32.2% in the glucose-based diet group and decreased by 24.3% in the lipid-based diet but the results were not statistically significant	[95]
Glioblastoma	20 patients with recurrent disease	KD (calories: 77% fat, 8% carbohydrates, and 15% protein) for 3–9 months in combination with temozolomide (TMZ) or chemoradiation	Four patients were alive at median follow-up of 14 months; one of the four patients was under carbohydrate-restricted KD (4.5% carbohydrates) post radiation and TMZ treatment and had no recurrence after 12 months from treatment; the other three had recurrence and started alternative chemotherapy treatments	[96]
Malignant disease	5 patients with severe weight loss	KD (70% MCT supplemented with *β*-hydroxybutyrate (BHB))	Increased body weight after 7 days (∼2 kg), presence of ketosis already after 24 h in association with a reduction of blood glucose, pyruvate, and lactate levels	[97]

## Abbreviations

OXPHOS: Oxidative phosphorylation
ATP: Adenosine triphosphate
NAD: Nicotinamide adenine dinucleotide
ROS: Reactive oxygen species
FDG-PET: F-18 fluorodeoxyglucose-positron emission tomography
GLUT: Glucose transporter
2-DG: 2-Deoxy-D-glucose
DCA: Dichloroacetate
3-BP: 3-Bromopyruvate
CR: Calorie restriction
IGF-1: Insulin-like growth factor-1
PI3K: Phosphatidylinositol-3 kinase
PKB: Protein kinase B
mTORC1: Mammalian target of rapamycin complex 1
Nrf2: Nuclear factor erythroid 2-related factor 2
AMPK: Adenosine monophosphate-activated protein kinase
PPAR*α*: Peroxisome proliferator-activated receptor-alpha
PGC1*α*: Peroxisome proliferator-activated receptor gamma coactivator 1-alpha
KD: Ketogenic diet.
